# Intercomparison of methods of coupling between convection and large‐scale circulation: 1. Comparison over uniform surface conditions

**DOI:** 10.1002/2015MS000468

**Published:** 2015-10-24

**Authors:** C. L. Daleu, R. S. Plant, S. J. Woolnough, S. Sessions, M. J. Herman, A. Sobel, S. Wang, D. Kim, A. Cheng, G. Bellon, P. Peyrille, F. Ferry, P. Siebesma, L. van Ulft

**Affiliations:** ^1^ Department of MeteorologyUniversity of ReadingReadingUK; ^2^ National Centre for Atmospheric Science, Department of MeteorologyUniversity of ReadingReadingUK; ^3^ Department of PhysicsNew Mexico TechSocorroNew MexicoUSA; ^4^ Department of Environmental SciencesColumbia UniversityNew YorkNew YorkUSA; ^5^ Department of Applied Physics and Applied MathematicsColumbia UniversityNew YorkNew YorkUSA; ^6^ Department of Atmospheric SciencesUniversity of WashingtonSeattleWashingtonUSA; ^7^ Climate Science Branch, NASA Langley Research CentreHamptonVirginiaUSA; ^8^ The Department of PhysicsUniversity of AucklandAucklandNew Zealand; ^9^ Meteo FranceToulouseFrance; ^10^ Royal Netherlands Meteorological InstituteDe BiltNetherlands; ^11^ Delft University of TechnologyDelftNetherlands

**Keywords:** tropical convection, large‐scale parameterized dynamics, weak‐temperature gradient, damped gravity wave, multiple equilibria

## Abstract

As part of an international intercomparison project, a set of single‐column models (SCMs) and cloud‐resolving models (CRMs) are run under the weak‐temperature gradient (WTG) method and the damped gravity wave (DGW) method. For each model, the implementation of the WTG or DGW method involves a simulated column which is coupled to a reference state defined with profiles obtained from the same model in radiative‐convective equilibrium. The simulated column has the same surface conditions as the reference state and is initialized with profiles from the reference state. We performed systematic comparison of the behavior of different models under a consistent implementation of the WTG method and the DGW method and systematic comparison of the WTG and DGW methods in models with different physics and numerics. CRMs and SCMs produce a variety of behaviors under both WTG and DGW methods. Some of the models reproduce the reference state while others sustain a large‐scale circulation which results in either substantially lower or higher precipitation compared to the value of the reference state. CRMs show a fairly linear relationship between precipitation and circulation strength. SCMs display a wider range of behaviors than CRMs. Some SCMs under the WTG method produce zero precipitation. Within an individual SCM, a DGW simulation and a corresponding WTG simulation can produce different signed circulation. When initialized with a dry troposphere, DGW simulations always result in a precipitating equilibrium state. The greatest sensitivities to the initial moisture conditions occur for multiple stable equilibria in some WTG simulations, corresponding to either a dry equilibrium state when initialized as dry or a precipitating equilibrium state when initialized as moist. Multiple equilibria are seen in more WTG simulations for higher SST. In some models, the existence of multiple equilibria is sensitive to some parameters in the WTG calculations.

## Key Points

- SCMs under both WTG and DGW methods display a much wider range of behaviors compared to CRMs
- DGW simulations always result in a precipitating equilibrium states
- Multiple stable equilibria exist in some WTG simulations

## 1 Introduction

The two‐way interaction between tropical deep convection and large‐scale tropical dynamics is a key issue in understanding tropical climate and its variability. In the past decade, this issue has been studied at a reasonable computational cost in both single‐column models (SCMs) and cloud‐resolving models (CRMs), using various forms of parameterized large‐scale dynamics. Parameterized large‐scale dynamics is a set of methods developed to capture the feedbacks of large‐scale tropical dynamics on convection, without explicitly simulating the large scale, based on a physical understanding of the tropical atmosphere.

One of the large‐scale parameterization methods, namely, the weak‐temperature gradient (WTG) approximation, has been used in many studies [e.g., *Sobel and Bretherton*, 2000; *Raymond and Zeng*, 2005; *Sobel et al*., 2007; *Sessions et al*., 2010; *Daleu et al*., 2012]. The WTG method relies on the physical principle that horizontal temperature gradients are very weak in the tropics, due to gravity waves which act to redistribute local buoyancy anomalies [*Bretherton and Smolarkiewcz*, 1989; *Mapes and Houze*, 1995; *Yano and Bonazzola*, 2009]. This method is valid only near the equator where the action of the Coriolis force is small, and in the tropical free troposphere at levels where the stratification allows such waves. *Sobel and Bretherton* [2000] made use of this physical principle to parameterize a large‐scale tropical circulation that consumes the simulated heating and accordingly maintains zero horizontal temperature gradient. Most subsequent WTG studies have imposed a weaker constraint in which the parameterized large‐scale circulation removes the horizontal temperature gradient over a short but nonzero time scale [e.g., *Shaevitz and Sobel*, 2004; *Raymond and Zeng*, 2005; *Sobel et al*., 2007; *Sessions et al*., 2010; *Wang and Sobel*, 2011; *Daleu et al*., 2012; *Wang et al*., 2013]. A recent innovation of this method is WTG simulations with spectral decomposition of heating in the vertical dimension [*Herman and Raymond*, 2014].

Another large‐scale parameterization method, namely, the damped gravity wave (DGW) method, derives the large‐scale vertical velocity directly from the approximated momentum equations. This parameterization method has been applied in several studies that simulate the two‐way coupling between convection and large‐scale dynamics, with the latter being simplified to a linear gravity wave of a single horizontal wave number [*Kuang*, 2008, 2011; *Wang et al*., 2013; *Romps*, 2012a, 2012b; *Edman and Romps*, 2015].

These two large‐scale parameterization methods (the WTG method and the DGW method) have proved to be useful frameworks that offer a pathway to attack the key question of what controls large‐scale variation of tropical deep convection. The configuration that is studied usually involves a reference reservoir column which is coupled to an interactive column simulated by a CRM or a SCM [e.g., *Raymond and Zeng*, 2005; *Sobel et al*., 2007; *Sessions et al*., 2010; *Wang and Sobel*, 2011; *Kuang*, 2008, 2011; *Wang and Sobel*, 2012; *Wang et al*., 2013; *Romps*, 2012a, 2012b]. Recently, however, *Daleu et al*. [2012] developed a new configuration that couples two interacting columns via a WTG derived large‐scale circulation to study the influence on local convection due to changes in remote convection [*Daleu et al*., 2014].

Much insight has been learned from these efforts. Unfortunately, many aspects of these large‐scale parameterization methods remain uncertain since the published results using these methods show both similarities and discrepancies in model behavior. An example of a discrepancy is found in an evaluation of the simulations of SCMs and CRMs with surface conditions identical to those of the reference column. In some studies, the equilibrium state obtained is almost identical to the state of the reference column [e.g., *Sobel and Bretherton*, 2000], while others obtained a simulated mean precipitation rate which is either greater than the implied value for the reference column [e.g., *Sobel et al*., 2007], or smaller than the implied value for the reference column [e.g., *Raymond and Zeng*, 2005; *Daleu et al*., 2012; *Herman and Raymond*, 2014].

Other examples of discrepancies are found in the evaluation of the shape of the derived large‐scale vertical velocity, the sensitivity of the simulated precipitation to changes in surface conditions, and the sensitivity of the final equilibrium state to the initial moisture conditions. The WTG method often produces large‐scale vertical velocities that are top‐heavy and not as smooth [e.g., *Raymond and Zeng*, 2005; *Sobel et al*., 2007; *Daleu et al*., 2012] as those obtained from the DGW method [e.g., *Kuang*, 2012; *Wang et al*., 2013]. *Romps* [2012a, 2012b] presented a particularly straightforward comparison of this attribute between the two schemes. For a given method of parameterization of the large‐scale circulation, some models are less sensitive to changes in surface conditions compared to others. Finally, while some models that parameterize the large‐scale circulation are not sensitive to the initial moisture conditions, others can sustain either an equilibrium state with persistent, precipitating convection or else an equilibrium state with zero precipitation depending on the initial moisture conditions [e.g., *Sobel et al*., 2007; *Sessions et al*., 2010; *Emanuel et al*., 2014].

In practice, the WTG method is not applied in the boundary layer. This requirement is respected by imposing a nominal boundary layer top below which the values of the large‐scale vertical velocities are calculated by the linear interpolation from the value diagnosed at the nominal top to the value of zero at the surface [e.g., *Sobel and Bretherton*, 2000; *Raymond and Zeng*, 2005; *Daleu et al*., 2012]. In addition, the WTG method performs poorly at levels where the static stability is close to zero [e.g., *Raymond and Zeng*, 2005; *Daleu et al*., 2012]. This problem is commonly resolved by imposing a lower bound to the static stability used to calculate the WTG vertical velocity [e.g., *Raymond and Zeng*, 2005; *Sessions et al*., 2010; *Daleu et al*., 2012]. Given these two required fixes in addition to the fact that the WTG method derives the large‐scale vertical velocity from the buoyancy anomalies rather than from the momentum equations as in the DGW method, it may appear that the WTG method is a less appropriate approach to capture the relevant dynamics, and the nature of the results may be sensitive to the details of its implementation. Caveats arise in the use of the DGW method as well, since it has its own assumptions. In particular, it considers one horizontal wave number at a time and assumes a simplified form of damping which requires an extra parameter besides the gravity wave horizontal length scale. Previous direct comparisons between the WTG and DGW schemes are found in *Romps* [2012a, 2012b], *Edman and Romps* [2014], and *Edman and Romps* [2015].

However, it should also be recognized that various other factors are important for the evolution of convective cells and thus, the results of their interactions with large‐scale dynamics. These include model physics, geometry [e.g., *Bretherton and Smolarkiewcz*, 1989; *Tompkins*, 2000; *Petch et al*., 2008], horizontal domain size [e.g., *Tompkins*, 2000; *Bretherton and Smolarkiewcz*, 1989], horizontal resolution [e.g., *Bryan et al*., 2003], and cloud‐radiative feedbacks [e.g., *Held et al*., 1993; *Tompkins and Craig*, 1998]. Therefore, some of the discrepancies between different studies that are seen in the published results may simply be the result of model dependency or different model setups.

It is clear that differences in the published results may be the result of the choice of large‐scale parameterization method and its implementation or may be the result of differences of the cloud model physics used to simulate convection. The Global Energy and Water Exchanges (GEWEX) Global Atmospheric Systems Modelling Panel (GASS) developed this international intercomparison project, the GASS‐WTG project, to develop community understanding of the large‐scale parameterization methods currently in use, to identify differences in behavior of different SCMs to inform parameterization development, and to assess the usefulness of these approaches as tool for parameterization development. As part of this project, we perform systematic comparisons of the WTG and DGW methods with a consistent implementation in a number of CRMs and SCMs, and systematic comparison of the behavior of CRMs and SCMs under the WTG method and DGW method. Part 1 of this study considers the case of equivalent surface conditions between the simulated column and the reference column, while Part 2 will focus on the sensitivity to SST in the simulated column. Part 1 is organized as follows. Section 2.1 describes the models that have contributed to this study. Section 2.2 presents the radiative‐convective equilibrium states that are used to define the reference states and to provide a set of initial conditions for the simulations with parameterized large‐scale circulation. Section 3 details our implementation of the WTG and DGW methods. Section 4 compares the results of the WTG and DGW simulations over uniform SST, including the results from the sensitivity to initial moisture conditions. Finally, the conclusions and the implications of our study are discussed in section 5.

## 2 Models Description and Radiative‐Convective Equilibrium Simulations

### 2.1 Models Description

Six groups participating in this intercomparison study performed simulations with 12 models. Five of these models are CRMs (two use three‐dimensions [3‐D] and three use two‐dimensions [2‐D]) while seven are SCMs. The models are listed in Tables 1 and 2 for CRMs and SCMs, respectively.

**Table 1:** List of Cloud‐Resolving Models (CRMs) That Participated in This Study^a^

	Cloud‐Resolving Models (CRMs)				
Model Type	Columbia University	CNRM‐GAME	NASA	New Mexico Tech	UK Met Office
Modelling Group					
Model ID	WRF	MesoNH	LaRC‐CRM	NMTCMv3	LEMv2.4
Symbol	•	▲	♦	▪	▼
Contributor	S. Wang	P. Peyrille	A. Cheng	M. J. Herman	C. Daleu
Country	U.S.	France	U.S.	U.S.	UK
Dimension	3‐D	3‐D	2‐D	2‐D	2‐D
Hor. size (km)	190 × 190	150 × 150	256	200	128
Hor. res (km)	2 × 2	3 × 3	4	1	0.5
No. of levels in the vertical	49	46	30	81	59

a The symbols serve as a legend for results presented in section 4.

**Table 2:** List of Single‐Column Models (SCMs) That Participated in This Study^a^

	Single‐Column Models (SCMs)						
Model Type	LMD/IPSL		NASA	CNRM‐GAME	UK Met Office	Royal Netherlands Meteorological Institute	
Modelling Group							
Model ID	LMDzA	LMDzB	GISS‐SCM	ARPEGEv6 (ARPv6)	UMv7.8	EC‐Earthv1	EC‐Earthv3
Symbol	◃	▹	°	▽	⋆	⋄	□
Contributor	G. Bellon	G. Bellon	D. Kim	G. Bellon	C. Daleu	B. van Ulft	B. van Ulft
Country	France	France	U.S.	France	UK	NL	NL
No. of levels in the vertical	39	39	40	91	63	61	61

a The symbols serve as a legend for results presented in section 4.

#### 2.1.1 Clouds Resolving Models

The Weather Research and Forecast (WRF) model version 3.3 [*Skamarock et al*., 2008] is configured in the WTG and DGW mode [*Wang and Sobel*, 2012; *Anber et al*., 2014]. Microphysics scheme is the Purdue‐Lin bulk scheme [*Lin et al*., 1983; *Rutledge and Hobbs*, 1984]. This scheme has six species: water vapor, cloud water, cloud ice, rain, snow, and graupel. The 2‐D Smagorinsky first‐order closure scheme is used to parameterize the horizontal transports by subgrid eddies. The surface fluxes of moisture and heat are parameterized following Monin‐Obukhov similarity theory. The Yonsei University (YSU) first‐order closure scheme is used to parameterize boundary layer turbulence and vertical subgrid scale eddy diffusion [*Hong and Pan*, 1996; *Noh et al*., 2003; *Hong et al*., 2006].

The mesoscale, nonhydrostatic atmospheric model MesoNH is described in *Lafore et al*. [1997]. The structure and evolution of the boundary layer is determined with a 1‐D eddy diffusivity turbulent scheme with a 1.5‐order closure for prognostic turbulent kinetic energy [*Cuxart et al*., 2000]. Thermals and shallow convection are parameterized with a mass flux approach from *Pergaud et al*. [2009]. The cloud microphysics are described by a mixed‐phase scheme [*Caniaux et al*., 1994; *Pinty and Jabouille*, 1998] that takes into account six water variables (water vapor, cloud droplets, raindrops, pristine ice, snow, and graupel). Surface fluxes are determined over the ocean from an iterative method based on *Belamari* [2005] and *Weill et al*. [2003].

The Langley Research Centre Cloud‐Resolving Model (LaRC‐CRM) is described in *Cheng and Xu* [2006]. It uses the analytical double‐Gaussian II probability distribution function proposed by *Larson et al*. [2002] to derive the cloud fraction and liquid water, and the buoyancy production terms of the second‐order and third‐order moment equations.

The New Mexico Tech cloud model is a toy model introduced in *Raymond and Zeng* [2005], with modifications and enhancements described in *Herman and Raymond* [2014]. A complete model description is found in the appendix of the latter work. The prognostic variables are specific moist entropy, total water mixing ratio (advected condensate and water vapor), rainfall mixing ratio, and momentum. The model is fully compressible with Smagorinsky turbulent mixing, bulk surface fluxes and a simplified microphysics scheme. An approximated ideal gas law is used such that water loading is not considered.

The Met Office Large Eddy Model at version 2.4 is described in *Shutts and Gray* [1994] and *Petch and Gray* [2001]. It includes a five‐category prognostic microphysical scheme [*Swann*, 1998; *Brown and Heymsfield*, 2001] with prognostic variables for the mixing ratios of cloud water, rain, ice, graupel, and snow, and for the number concentrations of ice, graupel, and snow. The subgrid turbulence scheme is based on the first‐order SmagorinskyLilly approach [*Brown et al*., 1994].

#### 2.1.2 Single‐Column Models

LMDzA and LMDzB are the SCM versions of the atmospheric components of IPSL‐CM5A and IPSL‐CM5B [*Dufresne et al*., 2013]. In LMDzA, convection is parameterized by the *Emanuel*'s [1991] mass flux scheme where closure and triggering take into account both tropospheric instability and convective inhibition. The statistical cloud scheme follows *Bony and Emanuel* [2001]. LMDzB shows a new set of physical parameterizations including representations of boundary layer thermal plumes and of cold pools. Deep convection triggering and closure of deep convection are controlled by lifting due to these subgrid processes [*Hourdin et al*., 2013].

GISS‐SCM is the single‐column form of the post‐CMIP5 version of the National Aeronautics and Space Administration Goddard Institute for Space Studies (GISS) GCM Model E2. This version is an updated from the one used in CMIP5 [*Schmidt et al*., 2014]. The cumulus [*Kim et al*., 2012] and planetary boundary layer parameterizations [*Yao and Cheng*, 2012] have been changed from the CMIP5 version, which led Model E2 to simulate a much better Madden‐Julian oscillation and slightly improved marine stratocumulus.

ARPEGE is the SCM version of the atmospheric component of the CNRM‐CM5 model [*Voldoire et al*., 2013]. Convection is parameterized by a mass flux scheme in which triggering depends on atmospheric stability and the closure is a function of moisture convergence [*Bougeault*, 1985]. A statistical cloud scheme developed by *Ricard and Royer* [1993] is included. This study uses a more recent version of the same model; ARPEGE version 6.04 (ARPv6) which has a buoyancy‐based parameterization of convection that includes prognostic condensates and improved convective transport (PCMT, Prognostic Condensates Microphysics and Transport) [*Piriou et al*., 2007; *Guérémy*, 2011].

UMv7.8 is the single‐column form of the Met Office Unified Model [*Davies et al*., 2005]. The convection parameterization is based on the bulk mass flux approach of *Gregory and Rowntree* [1990], with various subsequent modifications being described by *Derbyshire et al*. [2011], including an adaptive detrainment specification. Stratiform clouds are represented using the prognostic PC2 scheme of *Wilson et al*. [2008] with the associated microphysics following *Wilson and Ballard* [1999]. The boundary layer parameterization is that of *Lock et al*. [2000].

The SCM version of EC‐Earthv1 is based on the atmospheric circulation model IFS, cycle 31r1 of the European Centre for Medium‐Range Weather Forecasts (ECMWF) [*Hazeleger et al*., 2010]. Convection is based on a bulk mass flux scheme proposed by *Tiedtke* [1989] with updates described in *Bechtold et al*. [2004]. A cloud scheme with prognostic cloud water and cloud fraction developed described in *Tiedtke* [1993] is used. The boundary layer turbulence for convective conditions is parameterized by a combined Eddy‐Diffusivity Mass Flux (EDMF) approach [*Köhler et al*., 2011]. The SCM version of EC‐Earthv3 is based on IFS, cycle 36r4. The main relevant differences with version v1 are the introduction of a humidity‐dependent entrainment formulation [*Bechtold et al*., 2008] and the introduction of prognostic ice and rain water.

#### 2.1.3 Overall Approach

The lateral boundary conditions are periodic for all prognostic variables in all CRMs. To avoid the development of along‐domain wind shear that may occur [*Tompkins*, 2000; *Mapes and Wu*, 2001] and encourage the formation of squall lines [*Robe and Emanuel*, 2001; *Tao et al*., 1999] for the CRMs in 2‐D, the domain‐mean wind speeds in the along‐domain direction and in the across‐domain direction are relaxed toward vertically uniform values of 0 and 5 m s^−1^, respectively; both with a relaxation time scale of 6 h. For the purpose of fair comparison between 2‐D and 3‐D simulations, the horizontal domain‐mean wind speed components in the 3‐D models are relaxed toward vertically uniform values of 0 and 5 m s^−1^. The horizontal domain‐mean wind speed components in the SCMs are also relaxed toward vertically uniform values of 0 and 5 m s^−1^. Applying a vertically uniform wind speed of 5 m s^−1^ does not affect the dynamics of convection. It is simply used to increase the value of surface evaporation compared to the no wind value.

In all models, we use a spatially uniform and time‐independent sea surface temperature (SST) as the lower boundary condition and no Coriolis force is applied. Aside from any large‐scale circulation that might develop via the WTG or DGW method, these models are forced using an idealized cooling profile roughly approximating the effects of longwave radiation on the tropical troposphere. Such a cooling will henceforth be referred to as radiative cooling. It cools the temperature at a constant rate of 1.5 K d^−1^ from the surface to 200 hPa while maintaining the temperature of the upper troposphere and stratosphere at a uniform value of 200 K. Thus, the tendency of temperature due to radiative cooling, ${\left(\partial T/\partial t\right)}_{RC}$ is written as

(1) $${\left(\frac{\partial T}{\partial t}\right)}_{RC}=\{\begin{array}{ll} -1.5 & \;\text{if}\;\;\bar{p}\geq 200\\ -1.5\left(\frac{\bar{p}-100}{100}\right)-\alpha_{T}\left(\frac{200-\bar{p}}{100}\right)(\bar{T}-200) & \;\text{if}\;\;100<\bar{p}<200\\ -\alpha_{T}(\bar{T}-200) & \;\text{if}\;\;\bar{p}\leq 100 \end{array}$$

where the overbar denotes a horizontal domain‐average, *p* is the pressure in hPa, and $\alpha_{T}^{-1}=1$ day is the relaxation time scale of the temperature *T* toward a fixed value of 200 K at levels with $\bar{p}<100$ hPa. This treatment of radiative cooling is similar to that of *Pauluis and Garner* [2006] and *Wang and Sobel* [2011] for example. It produces a horizontally homogeneous and noninteractive cooling throughout most of the troposphere and hence, does not permit complications that may arise from radiative‐convective instability such as convective organization [*Held et al*., 1993; *Tompkins and Craig*, 1998]. The main focus of this study is the interactions between convection and large‐scale dynamics. Hence, the choice of using an idealized radiative cooling profile is made for simplicity only, and experiments to assess sensitivities to cloud‐radiation interactions are left to a future study.

### 2.2 Radiative‐Convective Equilibrium Simulations

To provide a reference profile which is consistent with the equilibrium state of each the models, we perform radiative‐convective equilibrium (RCE) simulations with the values of SST equal to 298, 300, and 302 K. The RCE simulations are run for a minimum period of 50 days, enough for each model to produce a quasi‐equilibrium state in which precipitation balances surface evaporation and the sensible and latent heat fluxes balance the radiative cooling.

**Figure 1:**
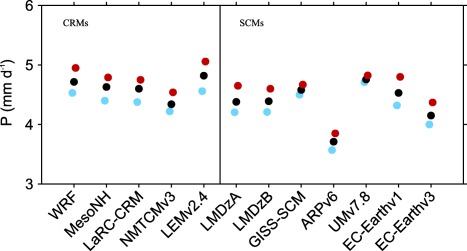
Mean precipitation rates at equilibrium. Results are obtained in the RCE simulations over an SST of 298 K (light blue), 300 K (black), and 302 K (red). The CRM and SCM results are shown on the left‐hand and right‐hand sides of the vertical line, respectively.

Figure 1 shows the mean precipitation rates at equilibrium in the RCE simulations of each of the models listed in Tables 1 and 2 with the SSTs of 298, 300, and 302 K. For all models, there is a slight increase in mean precipitation rate with SST. The increase in precipitation rate with SST is consistent with an increasing contribution from evaporation with increasing SST to the surface energy flux required to balance the radiative cooling.

**Figure 2:**
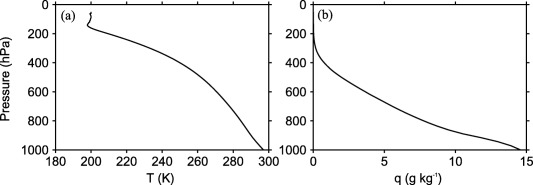
Profiles of (a) temperature and (b) specific humidity. Results are obtained by averaging RCE profiles of all CRMs. Results are shown for the RCE simulations with an SST of 300 K.

**Figure 3:**
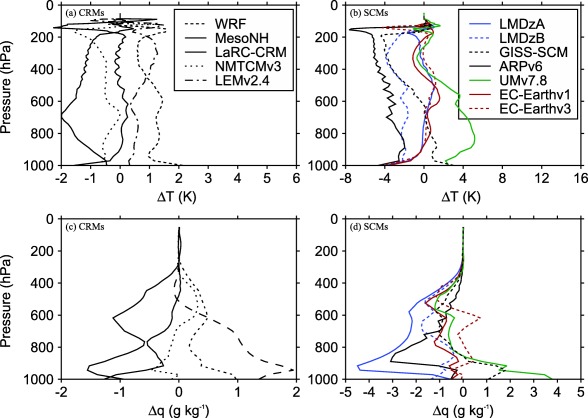
Difference in (a, b) temperature and (c, d) specific humidity between the RCE profiles of each of the models listed in Tables 1 and 2 and the profiles obtained by averaging over all CRMs. Results are those obtained in the (Figures 3a and 3c) CRMs and (Figures 3b and 3d) SCMs in a state of RCE over an SST of 300 K.

Figure 2 shows the mean profiles of temperature and humidity obtained by averaging the RCE profiles of all CRMs. Figure 3 shows the differences in temperature and humidity between the RCE profiles in each model compared to the profiles obtained by averaging over all CRMs (profiles shown in Figure 2). Results are shown for the RCE simulations over an SST of 300 K. Note the difference in the range of temperature and moisture profile differences for CRMs and SCMs. The moisture profile differences among CRMs are less than 2 g kg^−1^ in the boundary layer and less than 1 g kg^−1^ in the free troposphere. The temperature profile differences among CRMs are within 2 K throughout the column. However, there is a large spread among SCMs with a maximum moisture difference over 4 g kg^−1^ and a maximum temperature difference over 7 K. The shapes of temperature profiles are roughly similar. In all cases, temperature gradually decreases with pressure up to the first model level just above 200 hPa and then relaxes toward the fixed value of 200 K around 100 hPa (results not shown). Most models have similar static stability profiles except MesoNH, UMv7.8, and GISS‐SCM.

For all SSTs, the value of temperature at the model level just below 200 hPa differs from model to model, with the smallest value produced by ARPv6. As a result, ARPv6 produces the smallest radiative cooling (see equation (1)) compared to all other models. Since the radiative cooling rate is constant below 200 hPa, ARPv6 also produces the smallest column‐integrated radiative cooling rate. The value of surface sensible heat flux differs between models (results not shown) but is much smaller than surface latent heat flux. Thus, the main balance in RCE is between the column‐integrated radiative cooling rate and precipitation rate. Hence, models with weaker radiative cooling will generate less convective heating and therefore less precipitation than models with stronger radiative cooling. This is evident by noting that ARPv6 exhibits the smallest radiative cooling and produces the lowest precipitation rate (Figure 1). This flexibility in the value of column‐integrated radiative cooling rate combined with the flexibility in the value of surface sensible heat flux result in different values of precipitation rates when comparing models against each other. CRMs show less variation in mean precipitation rates compared to SCMs. UMv7.8 is much warmer and moister at the surface compared to all other models (see Figures 2b and 2d). However, since UMv7.8 produces values of surface fluxes which are very close to the values produced by other models, the analysis of the relationship between surface evaporation and moisture deficit reveals that UMv7.8 also has a much higher transfer coefficient compared to all other models (not shown). The RCE thermodynamic profiles are used to define the reference states in the implementation of the WTG and DGW methods, as well as providing initial conditions for the WTG and DGW simulations.

## 3 Parameterization of the Large‐Scale Dynamics

We use the WTG and DGW methods to parameterize large‐scale dynamics in the set of CRMs and SCMs listed in Tables 1 and 2. The WTG method relies on observations that in the deep tropics horizontal gradients of virtual potential temperature are small in the free troposphere. Assuming that the large‐scale dynamics act to maintain the domain‐mean virtual potential temperature close to a reference virtual potential temperature ${\bar{\theta }}_{v}^{Ref}$ in the free troposphere, the large‐scale pressure velocity, $\bar{\omega }$ is diagnosed there from the virtual potential temperature anomalies ${\bar{\theta }}_{v}-{\bar{\theta }}_{v}^{Ref}$ as

(2) $$\bar{\omega }\frac{\partial {\bar{\theta }}_{v}^{Ref}}{\partial p}=\frac{f(p)}{\tau }({\bar{\theta }}_{v}-{\bar{\theta }}_{v}^{Ref})$$

with the relaxation time scale *τ* = 3 h. The dimensionless function *f*(*p*) is introduced to allow the adjustment rate λ(p)=f(p)/τ to be function of pressure. The results presented in this paper are obtained from simulations performed with $f_{1}(p)=1$ as in *Wang and Sobel* [2011]. Some results from simulations performed with the half‐sine profile $f_{2}(p)=\sin(\pi (p_{s}-\bar{p})/(p_{s}-p_{t}))$ (as used in *Raymond and Zeng* [2005] and *Sessions et al*. [2010], with *p_s_* and *p_t_* the pressures at the surface and the tropopause, respectively) have also been obtained and these are mentioned in section 4.4.

In the boundary layer, surface fluxes create temperature gradients more efficiently than gravity waves damp them [*Sobel and Bretherton*, 2000]. In this study, the boundary layer is defined somewhat arbitrarily as those levels with pressures higher than or equal to *p_b_* = 850 hPa. We apply equation (2) from the first model level above *p_b_* to 100 hPa. Below *p_b_*, the values of $\bar{\omega }$ are obtained by linear interpolation in pressure from the value diagnosed at the first model level above *p_b_* to zero at the surface.

Equation (2) could produce very large and unphysical values of $\bar{\omega }$ if the static stability is very weak. To prevent our simulations from producing such values, we follow *Raymond and Zeng* [2005] and *Daleu et al*. [2012] and impose a lower bound equivalent to 1 K km^−1^ on the static stability when using equation (2).

The DGW method derives $\bar{\omega }$ from a wave equation that is obtained by combining the momentum and thermodynamics equations. This method has been used to allow the coupling between convection and large‐scale dynamics, which is simplified to a linear gravity wave of a single horizontal wave number [e.g., *Kuang*, 2008, 2011]. The DGW method relates the second‐order derivative of $\bar{\omega }$ to the virtual temperature anomalies $\bar{T_{v}}-{\bar{T}}_{v}^{Ref}$ as

(3) $$\frac{\partial }{\partial p}\left(\epsilon \frac{\partial \bar{\omega }}{\partial p}\right)=\frac{k^{2}R_{d}}{{\bar{p}}^{Ref}}({\bar{T}}_{v}-{\bar{T}}_{v}^{Ref})$$

where *ϵ* is the mechanical damping coefficient, *k* is the horizontal wave number, *R_d_* is the gas constant of dry air, ${\bar{T}}_{v}$ is the horizontal domain‐mean virtual temperature, and ${\bar{T}}_{v}^{Ref}$ is the target virtual temperature against which wave perturbations are computed. The elliptical equation (3) can be solved efficiently using a standard triangular matrix solver with boundary conditions $\bar{\omega }=0$ at the surface and at 100 hPa. A full description of the implementation of the DGW method used here is given in *Kuang* [2008, 2011]. Although some details of the implementation of the DGW method are different from the studies of *Romps* [2012a, 2012b], their common effect is to enforce a weak horizontal pressure gradient.

The parameters *τ* in the WTG method and *k* and *ϵ* in the DGW method are the key parameters that couple convection to the large‐scale motion and vice versa. In the WTG calculations, the same adjustment time scale, *τ* = 3 h, is used for all vertical modes. In the DGW calculations, we fix the value of *ϵ* = 1 day^−1^ and solve equation (3) with a single horizontal wave number $k=10^{-6}$ m^−1^. These are typical values used in previous WTG and DGW studies [e.g., *Herman and Raymond*, 2014; *Daleu et al*., 2012; *Wang and Sobel*, 2011; *Wang et al*., 2013]. In this study, the values of *τ*, *k*, and *ϵ* have been chosen such that the strength of the large‐scale circulations produced by a buoyancy anomaly with a first internal mode structure is comparable for the WTG and DGW methods. *Wang et al*. [2013] used the same values of *k* and *ϵ* and obtained a large‐scale circulation in a DGW simulation that was comparable in strength to that produced in a corresponding WTG simulation with the adjustment time scale of 4 h [see *Wang et al*., 2013, Figure 5]. The calculations of $\bar{\omega }$ given by equations (2) and (3) are performed either every 10 min (for models with integration time steps less than 10 min) or at every model time step (for models with larger time steps).

The large‐scale circulation parameterized in the model using either equation (2) or (3) introduces additional source and sink terms to the heat and moisture budgets. In this study, we consider its effects on potential temperature and water vapor only, so that the derived large‐scale circulation does not advect any other form of hydrometeor. Adiabatic heating or cooling of the column due to the derived large‐scale circulation, ${\left(\partial \theta /\partial t\right)}_{LS}$ is written as

(4) $${\left(\frac{\partial \theta }{\partial t}\right)}_{LS}=-\bar{\omega }\frac{\partial \bar{\theta }}{\partial p}$$

and the transport of moisture by the derived large‐scale circulation, ${\left(\partial q_{v}/\partial t\right)}_{LS}$ is written as

(5) $${\left(\frac{\partial q_{v}}{\partial t}\right)}_{LS}=-\bar{\omega }\frac{\partial {\bar{q}}_{v}}{\partial p}+\text{max}\left(\frac{\partial \bar{\omega }}{\partial p},0\right)({\bar{q}}_{v}^{Ref}-{\bar{q}}_{v})$$

where ${\bar{q}}_{v}$ is the domain‐mean specific humidity of water vapor and ${\bar{q}}_{v}^{Ref}$ is the specific humidity of the reference state. The term on the right‐hand side of equation (4) and the first term of the right‐hand side of equation (5) are the large‐scale vertical advection of potential temperature and water vapor, respectively. The second term on the right‐hand side of equation (5) is nonzero only if there is convergence into the simulated column. It represents the large‐scale horizontal advection of water vapor which in this study is parameterized as the drawing of the reference state air into the simulated domain by the diagnosed large‐scale circulation. It is described as “lateral entrainment” by *Raymond and Zeng* [2005] and used in many other studies [e.g., *Raymond and Sessions*, 2007; *Sessions et al*., 2010; *Wang et al*., 2013; *Herman and Raymond*, 2014]. However, it should be noted that other studies incorporated different representations of the horizontal moisture advection [e.g., *Sobel et al*., 2007; *Sobel and Bellon*, 2009; *Wang and Sobel*, 2012].

## 4 Results

For the implementation of the WTG and DGW methods, we need to prescribe the profiles of the reference state. For each model and for a given SST, the profiles of the reference state and the profiles used to initialize the WTG and DGW simulations are those obtained in the RCE simulation of the same model with the same SST.

We conducted a set of WTG and DGW simulations using each of the models listed in Tables 1 and 2. However, the time scale of adjustment of each model to a quasi‐equilibrium state with the parameterized large‐scale circulation is different and it is also different depending on which large‐scale parameterization method is used. Therefore, the simulations to be discussed were integrated over a period of time ranging between 50 and 250 days, and the mean states and statistics at equilibrium of each simulation have been obtained by averaging over a period of time such that a statistically steady state can be defined. The averaging period was the last 20 days in the 50 day simulations, 30 days in the 100 day simulations, and 100 days in the 250 day simulations.

### 4.1 Equilibrium State With Parameterized Large‐Scale Circulation

**Figure 4:**
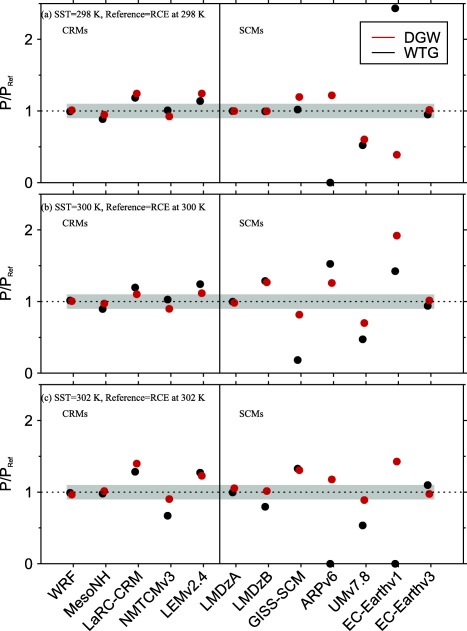
Ratios of mean precipitation rate of the simulated column P to the mean precipitation rate of the corresponding RCE reference state *P_Ref_*. Results are those obtained at equilibrium in the WTG (black circles) and DGW (red circles) simulations over an SST of (a) 298 K, (b) 300 K, and (c) 302 K. The CRM and SCM results are shown on the left‐hand and right‐hand sides of the vertical line, respectively. The grey area indicates $0.9<P/P_{Ref}<1.1$.

**Figure 5:**
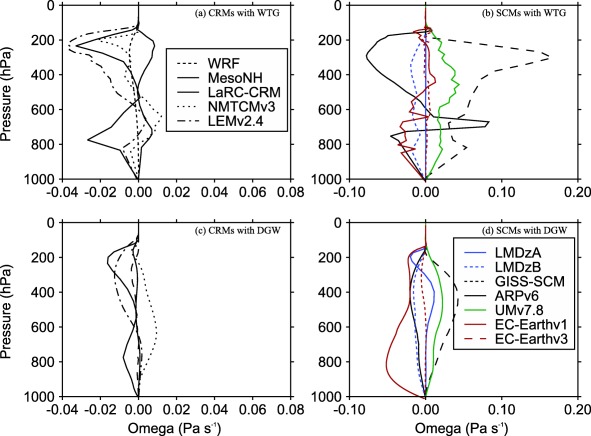
Large‐scale pressure velocities obtained at equilibrium in (top) the WTG and (bottom) DGW simulations over an SST of 300 K. Results are shown for the (left) CRMs and (right) SCMs. For each model, the reference profiles and the initial conditions are their own RCE profiles at 300 K.

We compared the equilibrium states produced in the WTG and DGW simulations to the RCE reference states using a ratio of mean precipitation rate of the simulated column, *P*, to the mean precipitation rate of the corresponding RCE reference state, *P_Ref_*, that is $P/P_{Ref}$. The values of $P/P_{Ref}$ are shown in the top, middle, and bottom of Figure 4 for *P* obtained at equilibrium in the WTG and DGW simulations over an SST of 298, 300, and 302 K, respectively. We also consider Figure 5, which shows the profiles of $\bar{\omega }$. These profiles are obtained at equilibrium in the WTG and DGW simulations performed over an SST of 300 K. For models in height coordinates, we expressed the large‐scale vertical velocities in Pa s^−1^ by applying the factor “ −ρg,” where *ρ* is density and *g* is the gravitational acceleration.

Since the simulated column in the WTG and DGW simulations has the same domain character (domain size, horizontal resolution, surface conditions, etc.) as the respective model's RCE reference state and is initialized with the profiles of the RCE reference state, the RCE reference state is a stable equilibrium state under the WTG and DGW configurations if the equilibrium state produced in the simulated column is similar to the RCE reference state. To provide a more quantitative evaluation of the simulations with parameterized large‐scale circulation, we calculated the mass‐weighted vertical integral of the large‐scale pressure velocities presented in Figure 5. That is $\Omega =\int \bar{\omega }dp/\Delta p$. Here we consider the derived large‐scale circulation to be negligible ( $\bar{\omega }\approx 0$) if $|\Omega |<0.4\times 10^{-2}$ Pa s^−1^ and the mean precipitation rate in the simulated column to be comparable to that in the RCE reference state if $0.9<P/P_{Ref}<1.1$. We chose *P* to be comparable to *P_Ref_* if it is within 10% of *P_Ref_* and for a typical value of column‐averaged static stability, our mass‐weighted large‐scale pressure velocity corresponds to column‐averaged large‐scale heating of about 10% of the radiative cooling rate imposed in the troposphere, and the two measures are self‐consistent.

**Figure 6:**
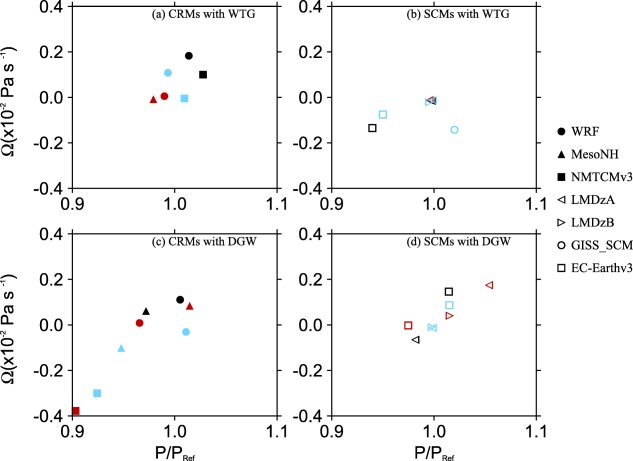
Scatterplots of Ω (the mass‐weighted vertical integral of the large‐scale pressure velocity) and $P/P_{Ref}$ (the ratio of mean precipitation rate of the simulated column to the mean precipitation rate of the RCE reference state). Results are shown for the (top) WTG and (bottom) DGW simulations over an SST of 298 K (light blue), 300 K (black), and 302 K (red). Results are shown for (left) CRMs and (right) SCMs. Results are shown for the WTG and DGW simulations which produce $|\Omega |<0.4\times 10^{-2}$ Pa s^−1^ and $0.9<P/P_{Ref}<1.1$.

The numerical values of Ω and $P/P_{Ref}$ are summarized in Tables 3 and 4 for CRMs and SCMs, respectively. Results in bold correspond to $|\Omega |<0.4\times 10^{-2}$ Pa s^−1^ or $0.9<P/P_{Ref}<1.1$. Figure 6 shows scatterplots of Ω and $P/P_{Ref}$ for the simulations which produce $|\Omega |<0.4\times 10^{-2}$ Pa s^−1^ and $0.9<P/P_{Ref}<1.1$. A model under the WTG or DGW method is considered to replicate the RCE conditions to a good approximation if the numerical values of Ω and $P/P_{Ref}$ are bold faced (Tables 3 and 4 insets) or if the corresponding symbol on the scatterplot of Ω versus $P/P_{Ref}$ is represented in Figure 6. Some models replicate the RCE reference state to a good approximation under both WTG and DGW, regardless of the SST. An example is WRF (light blue, black, and red solid circles in Figures 6a and 6c). Some models replicate the RCE reference state to a good approximation under either the WTG method or DGW method and for some SSTs only (e.g., NMTCMv3 under the WTG method; light blue and black solid squares in Figure 6a) and some do not reproduce the RCE reference state for any SST under any method (e.g., EC‐Earthv1; light blue, black, and red diamonds are not represented in Figures 6b and 6d). The DGW simulations are slightly more likely to reproduce the RCE reference state than the WTG simulations.

**Table 3:** Table Showing the Numerical Values of Ω (the Mass‐Weighted Vertical Integral of the Large‐Scale Pressure Velocity) and $P/P_{Ref}$ (the Ratio of Mean Precipitation Rate of the Simulated Column to the Mean Precipitation Rate of the RCE Reference State) for WTG and DGW Simulations Over a Uniform SST^a^

Model‐CRMs	WTG/DGW	SST (K)	$\Omega \times 10^{-2}$ Pa s^−1^	$P/P_{Ref}$
WRF	WTG	298	**0.110**	**0.990**
		300	**0.180**	**1.020**
		302	**0.005**	**0.987**
	DGW	298	**−0.031**	**1.010**
		300	**0.11**	**1.008**
		302	**0.009**	**0.960**
MesoNH	WTG	298	**−0.380**	0.886
		300	**−0.290**	0.896
		302	**−0.009**	**0.980**
	DGW	298	**−0.106**	**0.950**
		300	**0.060**	**0.970**
		302	**0.086**	**1.015**
LaRC‐CRM	WTG	298	1.150	1.180
		300	0.970	1.200
		302	1.240	1.280
	DGW	298	1.340	1.240
		300	0.610	1.102
		302	1.690	1.398
NMTCMv3	WTG	298	**0.005**	**1.009**
		300	**0.100**	**1.028**
		302	−1.320	0.670
	DGW	298	**−0.300**	**0.924**
		300	**−0.388**	0.896
		302	**−0.378**	**0.903**
LEMv2.4	WTG	298	0.650	1.140
		300	1.110	1.240
		302	1.560	1.270
	DGW	298	0.990	1.240
		300	0.464	1.117
		302	1.000	1.230

a These show results for the different CRMs. Results in bold correspond to $|\Omega |<0.4\times 10^{-2}$ Pa s^−1^ (or $\bar{\omega }\approx 0$) or $0.9<P/P_{Ref}<1.1$; if both columns are bold, the simulation with large‐scale parameterization reproduces the RCE state to a good approximation.

**Table 4:** Same as Table 3, but Lists SCM Results

Model‐SCMs	WTG/DGW	SST (K)	$\Omega \times 10^{-2}$ Pa s^−1^	$P/P_{Ref}$
LMDzA	WTG	298	**−0.013**	**0.998**
		300	**−0.015**	**0.997**
		302	**−0.013**	**0.990**
	DGW	298	**−0.013**	**0.998**
		300	**−0.065**	**0.982**
		302	**0.174**	**1.054**
LMDzB	WTG	298	**−0.021**	**0.998**
		300	1.180	1.290
		302	−0.780	0.790
	DGW	298	**−0.010**	**0.997**
		300	1.030	1.269
		302	**0.040**	**1.015**
GISS‐SCM	WTG	298	**−0.140**	**1.020**
		300	−5.700	0.180
		302	2.430	1.330
	DGW	298	1.880	1.195
		300	−2.180	0.820
		302	1.390	1.310
ARPv6	WTG	298	−7.490	0.000
		300	2.230	1.530
		302	−5.160	0.000
	DGW	298	0.970	1.220
		300	0.9720	1.260
		302	0.529	1.177
UMv7.8	WTG	298	−2.100	0.520
		300	−2.130	0.470
		302	−3.996	0.530
	DGW	298	−1.760	0.600
		300	−1.240	0.700
		302	−0.946	0.889
EC‐Earthv1	WTG	298	4.890	2.40
		300	0.990	1.420
		302	−3.980	0.000
	DGW	298	−1.750	0.390
		300	2.990	1.920
		302	1.440	1.430
EC‐Earthv3	WTG	298	**−0.075**	**0.950**
		300	**−0.1350**	**0.940**
		302	0.407	**1.098**
	DGW	298	**0.086**	**1.015**
		300	**1.146**	**1.014**
		302	**−0.002**	**0.975**

**Figure 7:**
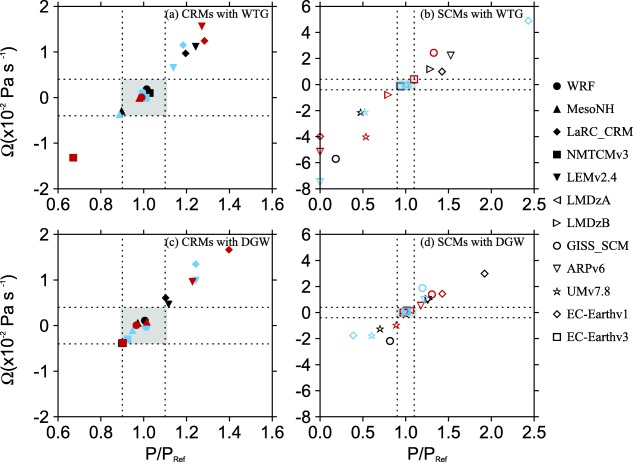
Similar to Figure 6, but results are shown for the WTG and DGW simulations of all the models listed in Tables 1 and 2 and for the SSTs of 298 K (light blue), 300 K (black), and 302 K (red). The grey boxes indicate $|\Omega |<0.4\times 10^{-2}$ Pa s^−1^ and $0.9<P/P_{Ref}<1.1$; shown in detail in Figure 6.

Figure 7 shows scatterplots of Ω and $P/P_{Ref}$ for the WTG and DGW simulations of all the models listed in Tables 1 and 2. Note the difference in axis for CRMs and SCMs. The grey areas in Figure 7 indicate $|\Omega |<0.4\times 10^{-2}$ Pa s^−1^ and $0.9<P/P_{Ref}<1.1$, shown in detail in Figure 6. A symbol which is outside the grey areas in Figure 7 corresponds to a model which, under the WTG or DGW method produces an equilibrium state which is significantly different from its RCE reference state (e.g., LEMv2.4 under the WTG method with an SST of 300 K; black solid inverted triangles in Figure 7a). For such WTG or DGW simulations, the equilibrium state produced in the simulated column is maintained by a large‐scale circulation established in the system.

The strength and direction of the circulation that develops over uniform SST differs from simulation to simulation, with a range of behaviors—including uniform ascent or descent, as well as layers of ascent and descent in the simulated column (see Figure 5). The simulations which produce uniform large‐scale ascent have an increase in mean precipitation rate relative to the value of the RCE reference state (e.g., the WTG simulation of LMDzB with an SST of 300 K; dashed blue curve in Figure 5b and black right facing triangle in Figure 7b) while those which produce uniform large‐scale descent have a decrease in mean precipitation rate relative to the value of the RCE reference state (e.g., the DGW simulation of UMv7.8 with an SST of 300 K; green curve in Figure 5d and black star in Figure 7d), consistent with the large‐scale moisture transports by the large‐scale circulation. In some SCMs under the WTG method, the large‐scale descent in the simulated column can be strong enough to inhibit precipitating convection completely. An example is the WTG simulation of EC‐Earthv1 with an SST of 302 K which shows $P/P_{Ref}=0$ in Figure 4c.

For the simulations which produce a large‐scale circulation with layers of both ascent and descent in the simulated column, the mean precipitation rate at equilibrium depends on the strength and the location of the ascending and descending branches. In some of those simulations, the enhancement or reduction of mean precipitation rate relative to the value of the RCE reference state follows the sign of the lower tropospheric circulation. For instance, the upper tropospheric descent and drying in the WTG simulation of EC‐Earthv1 with an SST of 300 K (solid red curve in Figure 5b) does not prevent an increase in precipitation rate because the lower tropospheric ascent has a net moistening effect as a consequence of vertical advection and lateral transport of moist environmental air near the boundary layer (equation (5)). Similarly, the upper tropospheric ascent and moistening in the DGW simulation of EC‐Earthv1 an SST of 298 K does not prevent a reduction in precipitation rate that is consequence of lower tropospheric descent and warming (large‐scale pressure velocity profile is not shown). In other simulations, the enhancement or reduction of precipitation rate relative to the value of the RCE reference state does not follow the sign of the lower tropospheric circulation. An example is the WTG simulation of LaRC‐CRM with an SST of 302 K, in which the lower tropospheric descent and drying is weak, so that an increase in precipitation rate occurs due to the strong middle and upper tropospheric ascent and moistening (large‐scale pressure velocity profile is not shown).

We compared the WTG and DGW results and the CRM and SCM results. Some models show sensitivity of the mean statistics (e.g., precipitation rates) to the SST which is not always monotonic (e.g., GISS‐SCM under the WTG method, Figure 4). Within the same SCM, a WTG simulation and a corresponding DGW simulation can produce different signs of the circulation, which suggest different characters of convection‐dynamics feedback. An example is EC‐Earthv1 with an SST of 298 K which produces $P/P_{Ref}>1.1$ under the WTG method and $P/P_{Ref}<0.9$ under the DGW method (EC‐Earthv1 in Figure 4a). The WTG method uses the same adjustment time scale for all the vertical modes and thus, damps the modes with shorter vertical wavelengths too quickly. In contrast, the DGW method damps the modes with shorter vertical wavelengths too slowly. Therefore, the couplings between convection and the large‐scale circulation on shorter vertical wavelengths are strengthened under the WTG method and weakened under the DGW method. These different effects on the damping rates of shorter vertical wavelengths produce the difference in smoothness between the large‐scale pressure velocity profiles obtained under the WTG and DGW methods [*Romps*, 2012b]; DGW simulations produce vertical velocity profiles which are generally smoother than those produced by WTG simulations (compare Figures 5b and 5d). There is a large spread among pressure velocities produced by SCMs compared to CRMs. Under the WTG method, for example, the large‐scale pressure velocity differences among SCMs range from −0.08 to 0.17 hPa s^−1^; which is much larger than the range of −0.04 to 0.01 hPa s^−1^ obtained among CRMs (compare Figures 5a and 5b). Finally, CRMs show a fairly linear relationship between Ω and *P*, with SCMs showing large deviations from this linear relationship particularly for simulations which produced strong descent and low precipitation (Figure 7). The relationship between precipitation and large‐scale circulation found in this study is qualitatively consistent with observations over the tropics; see for example *Oueslati and Bellon* [2013, Figure 11] which shows that over the tropics the relationship between large‐scale circulation and precipitation is close to linear in ascending regions and is not as linear in subsidence regions with reduced or zero precipitation.

### 4.2 Budget Analysis

Here we analyze the budgets in order to clarify the differences among RCE, WTG, and DGW simulations. The heat and moisture budgets for a simulation with parameterized large‐scale circulation are, respectively, written as

(6) $$H+P+R+C_{p}{〈\partial \bar{T}/\partial t〉}_{LS}=0\;and\;E-P+L_{v}{〈\partial \bar{q}/\partial t〉}_{LS}=0$$

with all variables written in energy units. The overbar indicates the domain and time‐average over a period of time when the statistically steady state is reached. *E*, *H*, *P*, and *R* denote the domain and time‐averaged values of surface evaporation, surface sensible heat flux, precipitation rate, and vertically integrated radiative cooling rate, respectively. The terms with angle brackets ( $〈\cdot 〉=\int_{p_{s}}^{p_{top}}\cdot dp/g$) in the heat and moisture budget equations represent the mean heating rate and moistening rate due to the diagnosed large‐scale circulation. They are hereafter denoted as *H_LS_* and *M_LS_*, respectively. *C_p_* is the heat capacity at constant pressure and *L_v_* is the latent heat of vaporization. For the RCE simulations, *H_LS_* and *M_LS_* are zero by definition.

**Figure 8:**
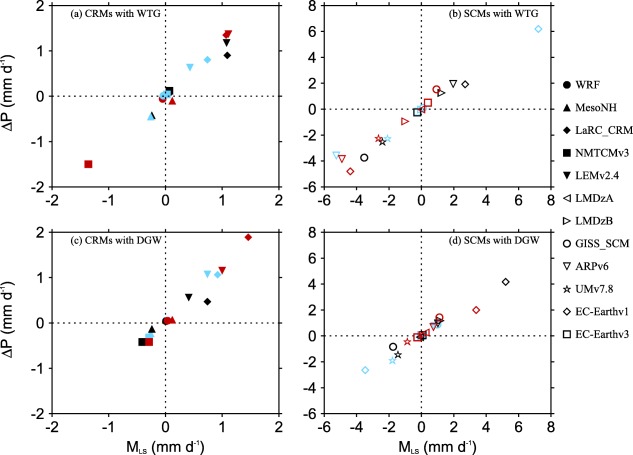
Scatterplots of ΔP (the changes in precipitation rate relative to the value of the RCE reference state) and *M_LS_* (the column‐integrated moistening rates due to the large‐scale circulation). The results are those obtained in the (top) WTG and (bottom) DGW simulations over an SST of 298 K (light blue), 300 K (black), and 302 K (red). Results are shown for (left) CRMs and (right) SCMs.

From the moisture budget equation, the changes in precipitation relative to the value of the RCE reference state, ΔP, must be due to either changes in surface evaporation, ΔE, or the moistening rate, *M_LS_*. Figure 8 shows scatterplots of ΔP against *M_LS_*. Both CRMs and SCMs show fairly linear relationships between ΔP and *M_LS_*. However, ΔP is not equal to *M_LS_* in most of the simulations, which implies changes in evaporation.

**Figure 9:**
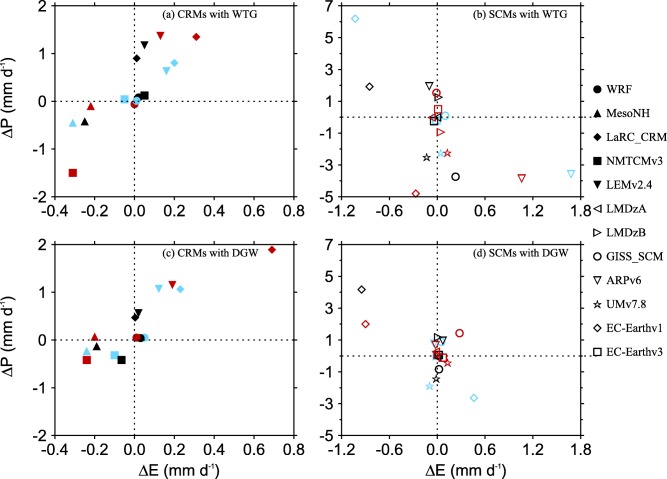
Scatterplots of ΔP (the changes in precipitation relative to the value of the RCE reference state) and ΔE (the changes in evaporation relative to the value of the RCE reference state). Results are those obtained in the (top) WTG and (bottom) DGW simulations over an SST of 298 K (light blue), 300 K (black), and 302 K (red). Results are shown for (left) CRMs and (right) SCMs.

Figure 9 shows scatterplots of ΔP against ΔE. Recall that this study imposes a mean horizontal wind in the surface flux calculations. As a result, the sensitivity of surface fluxes (sum of sensible heat and latent heat fluxes) to changes in near‐surface perturbation winds due to changes in convective activity is constrained. This is readily seen in Figure 9. ΔE is generally much smaller than ΔP, such that changes in precipitation are largely balanced by the moistening rates.

Despite the fact that the changes in surface fluxes have been constrained in this study, convective gustiness is more effective in CRMs than SCMs. For instance, ΔE increases with ΔP in a large proportion of CRM simulations while in SCM simulations, the enhancement of convective activity can be associated with a reduction in surface evaporation (e.g., the DGW simulation of EC‐Earthv1 with an SST of 300 K; black diamond in Figure 9d) or a suppression of convective activity can be associated with an increase in surface evaporation (e.g., the WTG simulation of ARPv6 with an SST of 302 K; red inverted triangle in Figure 9b) and there are many SCM simulations which produce zero changes in surface evaporation when convective activity is enhanced or suppressed (e.g., the WTG simulation of GISS‐SCM with an SST of 302 K; red circle in Figure 9b).

We now examine the relationship between the normalized gross moist stability (Γ) and changes in precipitation. *Raymond et al*. [2009] defined Γ as the dimensionless number which relates the net lateral outflow of moist static energy from a convective region to some measure of the strength of convection in that region. That is

(7) $$\Gamma =-〈\bar{\omega }\partial \bar{h}/\partial p〉/L〈\bar{\omega }\partial \bar{q}/\partial p〉$$

where *h* is the moist static energy, *q* is the specific humidity of water vapor, and *L* is the latent heat of vaporization.

However,

(8) $$〈\bar{\omega }\partial \bar{h}/\partial p〉=-H_{LS}-M_{LS}\;and\;〈\bar{\omega }\partial \bar{q}/\partial p〉=-M_{LS}$$

and from equations (6) and (8), a diagnostic equation for *P* is

(9) P−E=(E+H+R)/Γ

with

(10) $$\Gamma =-(M_{LS}+H_{LS})/M_{LS}$$

Equations similar to equation (9) have been used in previous studies to interpret convective responses to surface fluxes and radiative cooling [*Anber et al*., 2014] and external drying [*Wang and Sobel*, 2011].

**Figure 10:**
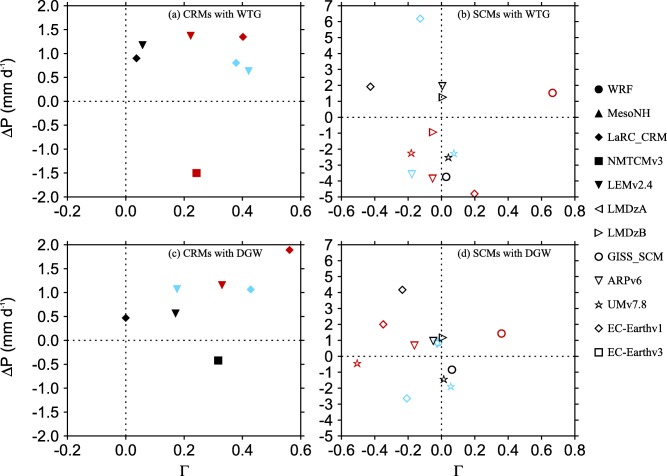
Scatterplots of ΔP (the changes in precipitation relative to the value of the RCE reference state) against Γ (the normalized gross moist stability). Results are shown for (left) CRMs and (right) SCMs. Results are shown for the (top) WTG and (bottom) DGW simulations over an SST of 298 K (light blue), 300 K (black), and 302 K (red) which result in significant large‐scale circulation ( $P/P_{Ref}<0.9$ or $P/P_{Ref}>1.1$ and $|\Omega |>0.4\times 10^{-2}$ Pa s^−1^). Results are shown for (left) CRMs and (right) SCMs.

We do not calculate Γ for WTG and DGW simulations which reproduce the RCE reference state to a good approximation, since Γ is a poor diagnostic when $M_{LS}+H_{LS}$ and *M_LS_* are both close to zero, consistent with a weak large‐scale circulation. Figure 10 shows scatterplots of ΔP against Γ for the simulations which produce significant large‐scale circulations at equilibrium ( $P/P_{Ref}<0.9$ or $P/P_{Ref}>1.1$ with $|\Omega |>0.4\times 10^{-2}$ Pa s^−1^). All CRMs with significant large‐scale circulation have positive values of Γ independent of the direction of the large‐scale circulation. Their values of Γ are less than 0.6 and the DGW simulation of LaRC‐CRM with an SST of 300 K is the only simulation which has Γ≈0 (black solid diamond in Figure 10c). In that simulation, *M_LS_* is nonzero and positive (see black solid diamond in Figure 8c), while $M_{LS}+H_{LS}\approx 0$ as the result of the net balance between the cooling and moistening rates. In contrast to CRMs, SCMs with significant large‐scale circulation can have positive or negative values of Γ independent of the direction of the large‐scale circulation (see Figures 10b and 10d).

In the absence of a large‐scale circulation P−E=0 and E+H+R=0. Hence, we can recast equation (9) in terms of the changes from the RCE values as

(11) $$\Delta P=\frac{\Gamma +1}{\Gamma }\Delta E+\frac{\Delta H+\Delta R}{\Gamma }$$

In this study, the sensitivity of radiative cooling to the changes in humidity and cloudiness has been constrained by imposing a fixed radiative cooling rate throughout most of the troposphere. As a result, ΔR is much smaller than ΔP for most of these simulations (results not shown). In addition, most of these simulations show that the sum of ΔH and ΔR is negligible compared to ΔE, which means that the factor (1+Γ)/Γ largely describes the strength of the relationship between ΔE and ΔP (see equation (11)). Figures 9 and 10 show that differences in both the gross moist stability and the changes in evaporation are important in determining the spread of precipitation changes observed in these models. Negative Γ (with |Γ|<1) as seen in some of the SCMs with significant large‐scale circulation means that ΔE and ΔP have opposite signs. For example, an increase in precipitation requires a reduction in evaporation as seen in the WTG simulation of EC‐Earthv1 with an SST of 300 K (black diamond in Figures 9b and 10b).

### 4.3 Sensitivity to Initial Moisture Conditions

We now examine the sensitivity of the final equilibrium state to the initial moisture conditions. We compare the equilibrium states produced with the simulated domain initialized with the relative humidity of the RCE reference state (i.e., the WTG and DGW simulations described above) to the equilibrium states produced in a set of parallel simulations with the simulated domain initialized with relative humidity equal to 0% at all model levels.

**Figure 11:**
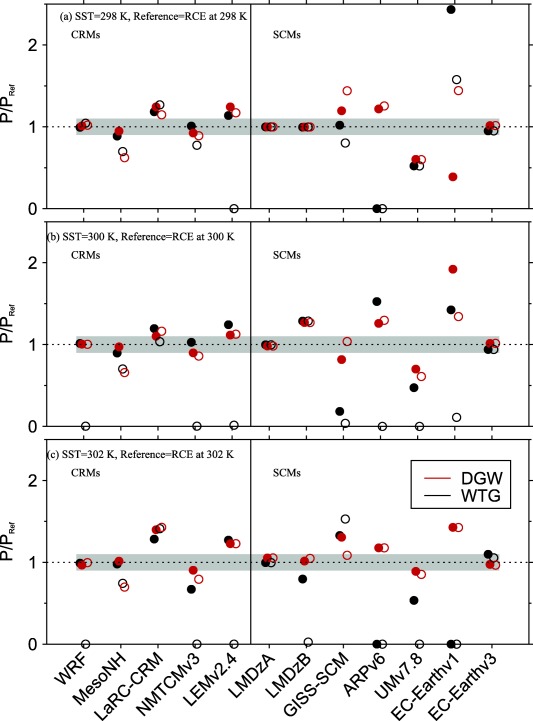
Ratios of mean precipitation rate of the simulated column P to the value of the corresponding RCE reference state *P_Ref_*. Results are obtained from the WTG (black circles) and DGW (red circles) simulations over an SST of (a) 298 K, (b) 300 K, and (c) 302 K. Results from the simulations initialized with the relative humidity from the RCE state (solid circles) are superimposed to the results from the simulations initialized with 0% relative humidity (circles). The CRM and SCM results are shown on the left‐hand and right‐hand sides of the vertical line, respectively. The grey area indicates $0.9<P/P_{Ref}<1.1$.

Figure 11 illustrates the dependence of *P* on the initial moisture conditions for each CRM and SCM. Initially, dry simulations are indicated by circles, while solid circles indicate initially moist simulations. DGW simulations always maintain a moist equilibrium state for any initial moisture condition (red circles and red solid circles in Figure 11 always show $P/P_{Ref}\neq 0$). Some DGW simulations produce precipitation rates that are independent of the initial moisture conditions (e.g., WRF with all SSTs). On the other hand, some DGW simulations produce precipitation rates that vary depending on the initial moisture (e.g., MesoNH with all SSTs), with some cases of increased precipitation rate from the completely dry initial conditions (e.g., EC‐Earthv1 with an SST of 298 K; Figure 11a).

WTG simulations (black circles and black solid circles in Figure 11) exhibit a wider range of outcome compared to DGW simulations. Unsurprisingly, simulations which produce zero precipitation when initialized with the relative humidity of the RCE reference state do not precipitate from the completely dry initial conditions. These include ARPv6 with SSTs of 298 and 302 K (Figures 11a and 11c), and EC‐Earthv1 with an SST of 302 K (Figure 11c). Some WTG simulations produce precipitation rates that are independent of the initial moisture conditions (e.g., LMDzA with all SSTs). As with the DGW simulations, some WTG simulations can sustain two distinct precipitating equilibrium states (e.g., MesoNH with all SSTs) but in contrast to DGW simulations, some WTG simulations can sustain either a persistent, precipitating convective state or a nonprecipitating state (hereafter called multiple equilibria), depending on the initial moisture conditions (e.g., LEMv2.4 with all SSTs). In some models, multiple equilibria under the WTG method are sustained for some SSTs only. Some examples are WRF with SSTs of 300 K or above (Figures 11b and 11c), LMDzB with SST of 302 K only (Figure 11c). Multiple equilibria are more obtained in WTG simulations with higher SST, although GISS‐SCM under the WTG method shows a nonmonotonic dependence on SST.

In all WTG simulations that sustain multiple equilibria, an initially moist column will sustain precipitating convection while an initially dry column will remain dry. The only difference in the simulations is the initial moisture profiles. In the initially dry state, there is a strong flux of moisture into the boundary layer from the sea surface. At the same time, a descending circulation is established in the simulated column as this cools without experiencing convective heating and also because $\theta_{v}-\theta_{v}^{Ref}<0$ when setting *q_v_* = 0 kg kg^−1^. Thus, to reach a precipitating state requires that sufficient moisture is supplied sufficiently quickly to develop precipitating convective cells in the presence of a subsidence warming and drying. This is consistent with the results of *Sessions et al*. [2010] and *Sobel et al*. [2007]. In such WTG simulations, a precipitating state is enabled when surface flux of moisture is sufficient to overcome subsidence drying. A useful further step would have been to determine the minimum initial relative humidity necessary to move the simulated column from the nonprecipitating state to the precipitating state [*Sessions et al*., 2010; *Emanuel et al*., 2014] or how much humidity the large‐scale circulation has to transport out of the simulated column in order to kill precipitating convection in the precipitating regime [*Sobel and Bellon*, 2009]. We do not address this issue in this study.

Large‐domain RCE simulations have shown the existence of both dry and moist regions due to self‐aggregation of convection, and cloud‐radiation interactions have been highlighted as one of the critical processes in the initiation of convective aggregation [*Bretherton et al*., 2005]. *Sessions et al*. [2010] and *Sobel et al*. [2007] performed small domain simulations with parameterized large‐scale circulation and interactive radiation. They demonstrated that a precipitating or nonprecipitating equilibrium states can be supported when very different moisture conditions are used. They related these multiple equilibrium states to the dry and moist regions obtained in large‐domain RCE simulations. However, our study uses noninteractive cooling throughout most of the troposphere and multiple equilibrium states similar to those obtained in *Sessions et al*. [2010] and *Sobel et al*. [2007] are nonetheless obtained in some of these models under the WTG method.

### 4.4 Multiple Equilibria and Sensitivity to WTG Parameters

**Figure 12:**
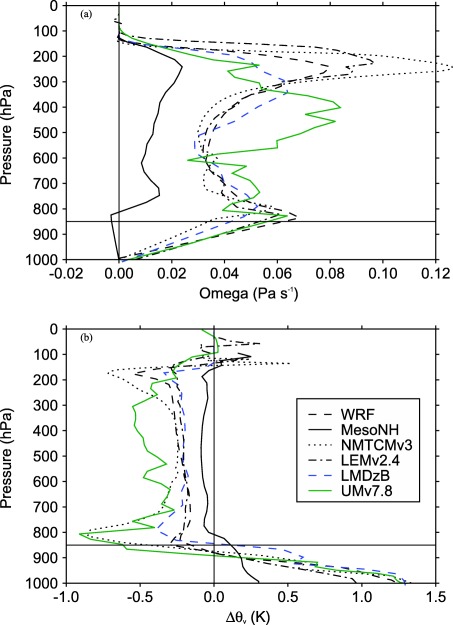
(top) Large‐scale pressure velocity and (bottom) deviation from the RCE reference profile of virtual potential temperature for the mean profile at equilibrium in the simulated column. Results are shown for the WTG simulations of MesoNH, NMTCMv3, LEMv2.4, WRF, LMDzB, and UMv7.8 with an SST of 302 K and with the initial relative humidity equals to 0% at all model levels.

From the studies of *Sessions et al*. [2010] and *Sobel et al*. [2007], multiple equilibria are sensitive to some parameters used in the implementation of the large‐scale parameterization method. In this section, we explore the ability of some models to sustain multiple equilibria. This is done by comparing the profiles obtained in the WTG simulations which produce a dry equilibrium state from the dry initial conditions to those obtained in the WTG simulations which produce a precipitating equilibrium state from the dry initial conditions. We find that in the simulations which produce a dry equilibrium state, the sign of the large‐scale circulation that is established below the boundary layer top *p_b_* is opposite to the sign of the circulation that would have been associated with the sign of ${\bar{\theta }}_{v}-{\bar{\theta }}_{v}^{Ref}$ there. Figure 12 shows some examples of $\bar{\omega }$ and ${\bar{\theta }}_{v}-{\bar{\theta }}_{v}^{Ref}$ profiles obtained at equilibrium in the WTG simulations performed over an SST of 302 K and with the simulated column initialized as completely dry. Results are shown for the WTG simulations which produce zero precipitation at equilibrium: NMTCMv3, LEMv2.4, WRF, LMDzB, and UMv7.8. They are compared to the results from the WTG simulation of MesoNH which produces a precipitating equilibrium state. In the WTG simulations with zero precipitation, the simulated column is drier (compared to the RCE reference state) throughout the column (results not shown). Hence, positive values of ${\bar{\theta }}_{v}-{\bar{\theta }}_{v}^{Ref}$ in the lower troposphere are the result of the simulated column being warmer than the RCE reference state. In all these simulations, there is a large‐scale descent in the middle and upper tropospheres, as would be expected from the negative sign of ${\bar{\theta }}_{v}-{\bar{\theta }}_{v}^{Ref}$. The boundary layer treatment used in the WTG calculations (section 3) implies that the sign of ${\bar{\theta }}_{v}-{\bar{\theta }}_{v}^{Ref}$ at the first model level above *p_b_* determines the sign of the large‐scale circulation below *p_b_*. As a result, there is large‐scale ascent below *p_b_* in the WTG simulation of MesoNH. In contrast, there is a large‐scale descent below *p_b_* in the WTG simulations of NMTCMv3, LEMv2.4, WRF, LMDzB, and UMv7.8. Positive values of ${\bar{\theta }}_{v}-{\bar{\theta }}_{v}^{Ref}$ below *p_b_* would correspond to large‐scale ascent without the special treatment of the boundary layer.

**Figure 13:**
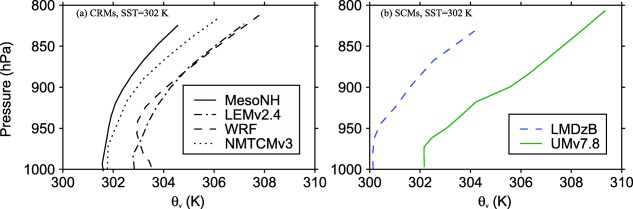
Profiles of virtual potential temperature in the lowest 200 hPa. Results are those obtained in the RCE simulations of (left) CRMs and (right) SCMs over an SST of 302 K.

**Figure 14:**
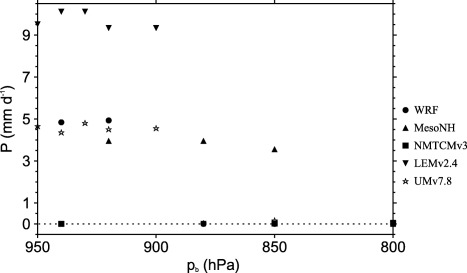
Mean precipitation rates as a function of the height of the nominal boundary layer used in the WTG calculations. Results are shown for the initially dry WTG simulations of MesoNH, LEMv2.4, WRF, NMTCMv3, and UMv7.8 with an SST of 302 K.

Figure 13 shows these models’ virtual potential temperature RCE profiles at 302 K in the lowest 200 hPa. For all of these models, the well mixed layer is less than 50 hPa and the value of *p_b_* = 850 hPa is clearly within the lower troposphere rather than being at the top of the boundary layer. Given the consequence of the boundary layer treatment used for the WTG calculations, a further step is to examine the sensitivity of the final equilibrium state to the nominal boundary layer depth *p_b_* (as was done in *Herman and Raymond* [2014]). We repeated some of the WTG simulations described above using *p_b_* = 800, 880, 900, 920, 930, 940, and 950 hPa. We discuss the results from the initially dry WTG simulations of LEMv2.4, UMv7.8, WRF, NMTCMv3, and MesoNH with an SST of 302 K. The mean precipitation rates obtained at equilibrium in the simulations with these values of *p_b_*, and in the simulations with *p_b_* = 850 hPa are plotted in Figure 14. The WTG simulations of NMTCMv3, LEMv2.4, WRF, LMDzB, and UMv7.8 with a deeper nominal boundary layer ( $p_{b}<850$ hPa) also produce positive values of ${\bar{\theta }}_{v}-{\bar{\theta }}_{v}^{Ref}$ below *p_b_* and negative values from the first model level above *p_b_* to the tropopause (not shown). As a result, the boundary layer treatment used in the WTG calculations forces a large‐scale descent throughout the column and equilibrium states with zero precipitation are achieved in those simulations. A similar result is obtained in the WTG simulation of MesoNH with *p_b_* = 800 hPa since, ${\bar{\theta }}_{v}-{\bar{\theta }}_{v}^{Ref}$ is negative at the first model level above 800 hPa.

A large‐scale descent below *p_b_* dries the boundary layer and may kill off precipitating convection. In contrast, a large‐scale ascent below *p_b_* favors precipitating convection as it moistens the boundary layer through vertical and horizontal advection. Results show that with the exception of the WTG simulation of NMTCMv3 which produces a nonprecipitating state even with *p_b_* = 950 hPa (solid squares in Figure 14), other WTG simulations have a critical nominal boundary layer depth below which the nonprecipitating state is destroyed due to the presence of large‐scale ascent in the boundary layer. Hence, a shallow nominal boundary layer can initiate precipitating convection and force the transition from the nonprecipitating state to the precipitating state. For instance, a precipitating state from the dry initial conditions is not obtained unless *p_b_* is increased to at least 850 hPa in the WTG simulation of MesoNH, 900 hPa in the WTG simulations of LEMv2.4 and UMv7.8 and 920 hPa in the WTG simulation of WRF. *Herman and Raymond* [2014] also demonstrated parameter sensitivity in multiple equilibrium experiments but they found an opposing trend in rain rate versus nominal boundary layer using NMTCMv3. The reasons for this discrepancy are as yet unclear.

These results demonstrate that the existence of multiple equilibria in some of the models under the WTG method depends on parameters in the implementation of the WTG method. In addition to the dependence on *p_b_*, we have examine the dependence on *f*(*p*) defined in section 3. The initially dry WTG simulations of LEMv2.4, NMTCMv3, and UMv7.8 with $f_{2}(p)$ and *τ* = 3 h produce precipitating equilibrium (results not shown) as opposed to the dry equilibrium obtained for $f_{1}(p)$. The half‐sine profile gives a short effective relaxation time scale in the middle troposphere and a long effective relaxation time scale in the lower and upper tropospheres. As shown in *Daleu et al*. [2012], the longer the relaxation time scale, the weaker the large‐scale circulation, and consistent with the results of *Sessions et al*. [2010], initially dry WTG simulations of LEMv2.4 and UMv7.8 with a long relaxation time scale (e.g., *τ* = 120 h and $f_{1}(p)$) produce equilibrium states with persistent, precipitating convection. This last result is also qualitatively consistent with the result of *Sobel et al*. [2007], who found that the nonprecipitating equilibrium state from the dry initial conditions is destroyed when longer relaxation times for horizontal moisture advection are used. However, in contrast to this study which considers horizontal moisture advection by the divergent circulation induced by enforcing WTG or DGW, *Sobel et al*. [2007] considered horizontal moisture advection by large‐scale rotational circulations and parameterized its effect by relaxing the domain‐mean moisture toward a reference profile over a time scale independent of the WTG adjustment time scale.

We have demonstrated the dependence of the existence of multiple equilibria in WTG simulations on both SST and the prescribed boundary layer depth. However, there are a range of further parameters including both model physics and experimental setup which may be important, but which have not been examined in this study.

## 5 Conclusions

In this international intercomparison project, we systematically compared the interactions between convection and large‐scale circulation in various CRMs and SCMs under two methods of parameterization of the large‐scale dynamics: the WTG method and the DGW method. The WTG method derives the large‐scale circulation from buoyancy anomalies with a given relaxation time scale [*Raymond and Zeng*, 2005; *Sobel et al*., 2007; *Sessions et al*., 2010; *Daleu et al*., 2012] while the DGW method derives the large‐scale circulation from the momentum equations [*Kuang*, 2008, 2011; *Romps*, 2012a, 2012b]. The derived large‐scale circulation couples a model to a reference state defined with profiles generated from previous RCE simulations of the same model. We conducted WTG and DGW simulations over uniform SSTs and compared the results from various CRMs and SCMs under each method.

When coupled to their own RCE profiles, some WTG and DGW simulations were able to reproduce their own reference solutions to a good approximation. Those simulations produce a negligible time‐mean large‐scale circulation and a mean precipitation rate which is very close to the value of their reference state. A similar result was produced in *Sobel and Bretherton* [2000], although other simulations from this study and previous studies [e.g., *Raymond and Zeng*, 2005; *Sobel et al*., 2007; *Sessions et al*., 2010] do not reproduce their reference conditions.

The WTG and DGW simulations which do not reproduce their reference conditions produce mean precipitation rates which differ substantially from the rates of their reference states; this difference is maintained by a large‐scale circulation in the system. In those simulations, the direction of the large‐scale circulation varies from uniform large‐scale ascent in the simulated column with a compensating increase in mean precipitation rate, uniform large‐scale descent in the simulated column with a compensating reduction in mean precipitation rate, and large‐scale circulation with layers of both ascent and descent in the simulated column. In the latter case, the changes in precipitation (relative to the value of the reference state) depend on the position and strength of the ascending and descending branches.

Some models under the WTG or DGW method produce large‐scale circulations of different magnitudes for different SSTs, and the sensitivity to the SSTs is not always monotonic. Some SCMs under the WTG method produce zero precipitation even with moist initial moisture conditions. Within the same SCM, a WTG simulation and a corresponding DGW simulation can produce large‐scale circulations of different signs. In general, DGW simulations produce large‐scale pressure velocity profiles which are smoother than those produced by WTG simulations.

When comparing SCMs and CRMs, we found a large spread among pressure velocities produced by SCMs compared to those produced by CRMs. CRMs show a fairly linear relationship between mean precipitation rates and the amplitude of the diagnosed large‐scale circulation, while SCMs show large deviations from this linear relationship particularly for simulations which produced strong descent and small precipitation rate.

An analysis of the heat, moisture, and moist static energy budgets shows that changes in precipitation are largely balanced by the moistening rates due to the large‐scale circulation. This is consistent with the smaller changes in surface evaporation, as a result of using fixed horizontal wind speed in the surface flux calculations. The analysis also shows that while all of the CRMs with significant large‐scale circulation produce positive values of normalized gross moist stability, some of the SCMs produce both positive and negative values of normalized gross moist stability independent of the direction of the large‐scale circulation.

In contrast to simulations using the DGW method, multiple equilibria corresponding to either a dry equilibrium state or a precipitating equilibrium state exist in some models under the WTG method. In all models that support multiple equilibria under the WTG method, an initially dry column remains dry for certain values of SST and its ability to develop and sustain precipitating convection is sensitive to some parameters in the WTG calculations. We have demonstrated the sensitivity of multiple equilibria to the nominal boundary layer depth, below which the large‐scale vertical velocities are calculated by linear interpolation from the value diagnosed at the specified boundary layer top to zero at the surface. A shallow nominal boundary layer (while nonetheless deeper than the models’ well mixed layer) can permit the development of large‐scale ascent in the lower troposphere and the associated column moistening necessary to initiate precipitating convection in some models. This finding contrasts with results of *Herman and Raymond* [2014], where in deeper nominal boundary layer favored a precipitating steady state. In three of the models, we have also explored the sensitivity of multiple equilibria to the relaxation time scale and the adjustment rate profile. A longer relaxation time scale throughout the column, or an adjustment rate profile which allows longer effective relaxation time scale in the lower troposphere, can also destroy the dry equilibrium solution. This sensitivity of multiple equilibria to the relaxation time scale is consistent with the result of *Sessions et al*. [2010]. It is also qualitatively similar to the results of *Sobel et al*. [2007], which showed the sensitivity of multiple equilibria to the time scale for moisture relaxation toward a given profile. Noting that multiple equilibria are seen in more CRMs and SCMs under the WTG method with a higher SST, we may conclude that SST (or surface wind speed which would modulate surface fluxes) is one of the factors which may control the existence of multiple equilibria.

This intercomparison project highlights some weaknesses of the large‐scale parameterization methods. Our results suggest that caution should be used when comparing results between different studies of this nature because the discrepancies between the published results can be related to differences in the physics of the convection models or the implementation of the large‐scale parameterization methods. For instance, some results from the WTG simulations are very sensitive to the details of the implementation of the WTG method.

The results from this intercomparison project are important not only for understanding the interactions between convection and large‐scale tropical dynamics but also for interpreting discrepancies between results reported in the literature. Models produce reasonable RCE states which are different when comparing models against each other. However, noting that different CRMs under parameterized large‐scale circulation behave broadly in the similar way while SCMs produce a much larger variation of behaviors, comparison between CRMs and SCMs behavior under parameterized large‐scale circulation may be a useful tool for trying to reduce biases or improve the SCMs or a useful tool when developing and testing parameterization schemes. Part 2 of this study will compare models and large‐scale parameterization methods over nonuniform surface conditions.

## References

[jame20217-bib-0001] Anber, U. , S. Wang , and A. Sobel (2014), Response of atmospheric convection to vertical wind shear: Cloud‐system‐resolving simulations with parameterized large‐scale circulation. Part I: Specified radiative cooling, J. Atmos. Sci., 71, 2976–2993.

[jame20217-bib-0002] Bechtold, P. , J.‐P. Chaboureau , A. Beljaars , A. Betts , M. Köhler , M. Miller , and J.‐L. Redelsperger (2004), The simulation of the diurnal cycle of convective precipitation over land in a global model, Q. J. R. Meteorol. Soc., 130, 3119–3137.

[jame20217-bib-0003] Bechtold, P. , M. Köhler , T. Jung , F. Doblas‐Reyes , M. Leutbecher , M. J. Rodwell , F. Vitart , and G. Balsamo (2008), Advances in simulating atmospheric variability with the ECMWF model: From synoptic to decadal time‐scales, Q. J. R. Meteorol. Soc., 134, 1337–1351.

[jame20217-bib-0004] Belamari, S. (2005), Report on uncertainty estimates of an optimal bulk formulation for surface turbulent fluxes, *MERSEA IP Deliverable D.4.1.2*, 29 pp.

[jame20217-bib-0005] Bony, S. , and K. Emanuel (2001), A parameterization of the cloudiness associated with cumulus convection; evaluation using TOGA COARE data, J. Atmos. Sci., 58, 3158–3183.

[jame20217-bib-0006] Bougeault, P. (1985), The diurnal cycle of the marine stratocumulus layer: A higher‐order model study, J. Atmos. Sci., 42, 2826–2843.

[jame20217-bib-0007] Bretherton, C. , and P. Smolarkiewcz (1989), Gravity waves, compensating subsidence and detrainment around cumulus clouds, J. Atmos. Sci., 46, 740–759.

[jame20217-bib-0008] Bretherton, C. , P. Blossey , and M. Khairoutdinov (2005), An energy‐balance analysis of deep convective self‐aggregation above uniform SST, J. Atmos. Sci., 62, 4273–4292.

[jame20217-bib-0009] Brown, A. , S. Derbyshire , and P. Mason (1994), Large‐eddy simulation of stable atmospheric boundary layers with a revised stochastic subgrid model, Q. J. R. Meteorol. Soc., 120, 1485–1512.

[jame20217-bib-0010] Brown, P. , and A. Heymsfield (2001), The microphysical properties of tropical convective anvil cirrus: A comparison of models and observations, Q. J. R. Meteorol. Soc., 127, 1535–1550.

[jame20217-bib-0011] Bryan, G. , J. Wyngaard , and J. Fritsch (2003), Resolution requirements for the simulation of deep moist convection, Mon. Weather Rev., 131, 2394–2416.

[jame20217-bib-0012] Caniaux, G. , J. Redelsperger , and J. Lafore (1994), A numerical study of the stratiform region of a fast‐moving squall line. Part I: General description and water and heat budgets, J. Atmos. Sci., 51, 2046–2074.

[jame20217-bib-0013] Cheng, A. , and K.‐M. Xu (2006), Simulation of shallow cumuli and their transition to deep convective clouds by cloud‐resolving models with different third‐order turbulence closures, Q. J. R. Meteorol. Soc., 132, 359–382.

[jame20217-bib-0014] Cuxart, J. , P. Bougeault , and J.‐L. Redelsperger (2000), A turbulence scheme allowing for mesoscale and large‐eddy simulations, Q. J. R. Meteorol. Soc., 126, 1–30.

[jame20217-bib-0015] Daleu, C. , S. Woolnough , and R. Plant (2012), Cloud‐resolving model simulations with one and two‐way couplings via the weak‐temperature gradient approximation, J. Atmos. Sci., 69, 3683–3699.

[jame20217-bib-0016] Daleu, C. , S. Woolnough , and R. Plant (2014), Transition from suppressed to active convection modulated by a weak‐temperature gradient derived large‐scale circulation, J. Atmos. Sci., 72, 834–853.

[jame20217-bib-0017] Davies, T. , M. Cullen , A. Malcolm , M. Mawson , A. Staniforth , A. White , and N. Wood (2005), A new dynamical core for the Met Office's global and regional modelling of the atmosphere, Q. J. R. Meteorol. Soc., 131, 1759–1782.

[jame20217-bib-0018] Derbyshire, S. , A. Maidens , S. Milton , R. Stratton , and M. Willett (2011), Adaptive detrainment in a convective parametrization, Q. J. R. Meteorol. Soc., 137, 1856–1871.

[jame20217-bib-0019] Dufresne, J.‐L. , et al. (2013), Climate change projections using the IPSL‐CM5 earth system model: From CMIP3 to CMIP5, Clim. Dyn., 40, 2123–2165.

[jame20217-bib-0020] Edman, J. P. , and D. M. Romps (2014), An improved weak pressure gradient scheme for single‐column modeling, J. Atmos. Sci., 71, 2415–2429.

[jame20217-bib-0021] Edman, J. P. , and D. M. Romps (2015), Self‐consistency tests of large‐scale‐dynamics parameterizations for single‐column modeling, J. Adv. Model. Earth. Syst., 7, 320–334, doi:10.1002/2014MS000378.

[jame20217-bib-0022] Emanuel, K. (1991), A scheme for representing cumulus convection in large‐scale models, J. Atmos. Sci., 48, 2313–2329.

[jame20217-bib-0023] Emanuel, K. , A. A. Wing , and E. M. Vincent (2014), Radiative‐convective instability, J. Adv. Model. Earth Syst., 6, 75–90, doi:10.1002/2013MS000270.

[jame20217-bib-0024] Gregory, D. , and P. R. Rowntree (1990), A mass flux convection scheme with representation of cloud ensemble characteristics and stability‐dependent closure, Mon. Weather. Rev., 118, 1483–1506.

[jame20217-bib-0025] Guérémy, J. (2011), A continuous buoyancy based convection scheme: One‐and three‐dimensional validation, Tellus, Ser. A, 63, 687–706.

[jame20217-bib-0026] Hazeleger, W. , et al. (2010), EC‐Earth: A seamless earth‐system prediction approach in action, Bull. Am. Meteorol. Soc., 91, 1357–1363.

[jame20217-bib-0027] Held, I. M. , R. S. Hemler , and V. Ramaswamy (1993), Radiative‐convective equilibrium with explicit two‐dimensional moist convection, J. Atmos. Sci., 50, 3909–3927.

[jame20217-bib-0028] Herman, M. J. , and D. J. Raymond (2014), WTG cloud modeling with spectral decomposition of heating, J. Adv. Model. Earth. Syst., 6, 1121–1140, doi:10.1002/2014MS000359.

[jame20217-bib-0029] Hong, S.‐Y. , and H.‐L. Pan (1996), Nonlocal boundary layer vertical diffusion in a medium‐range forecast model, Mon. Weather. Rev., 124, 2322–2339.

[jame20217-bib-0030] Hong, S.‐Y. , Y. Noh , and J. Dudhia (2006), A new vertical diffusion package with an explicit treatment of entrainment processes, Mon. Weather. Rev., 134, 2318–2341.

[jame20217-bib-0031] Hourdin, F. , et al. (2013), LMDz5b: The atmospheric component of the IPSL climate model with revisited parameterizations for clouds and convection, Clim. Dyn., 40, 2193–2222.

[jame20217-bib-0032] Kim, D. , A. H. Sobel , A. D. Del Genio , Y. Chen , S. J. Camargo , M.‐S. Yao , M. Kelley , and L. Nazarenko (2012), The tropical subseasonal variability simulated in the NASA GISS general circulation model, J. Clim., 25, 4641–4659.

[jame20217-bib-0033] Köhler, M. , M. Ahlgrimm , and A. Beljaars (2011), Unified treatment of dry convective and stratocumulus‐topped boundary layers in the ECMWF model, Q. J. R. Meteorol. Soc., 137, 43–57.

[jame20217-bib-0034] Kuang, Z. (2008), Modeling the interaction between cumulus convection and linear gravity waves using a limited‐domain cloud system‐resolving model, J. Atmos. Sci., 65, 576–591.

[jame20217-bib-0035] Kuang, Z. (2011), The wavelength dependence of the gross moist stability and the scale selection in the instability of column‐integrated moist static energy, J. Atmos. Sci., 68, 61–74.

[jame20217-bib-0036] Kuang, Z. (2012), Weakly forced mock Walker cells, J. Atmos. Sci., 69, 2759–2786.

[jame20217-bib-0037] Lafore, J. P. , et al. (1997), The Meso‐NH atmospheric simulation system. Part I: Adiabatic formulation and control simulations, Ann. Geophys., 16, 90–109.

[jame20217-bib-0038] Larson, V. E. , J.‐C. Golaz , and W. R. Cotton (2002), Small‐scale and mesoscale variability in cloudy boundary layers: Joint probability density functions, J. Atmos. Sci., 59, 3519–3539.

[jame20217-bib-0039] Lin, Y. , R. Farley , and H. Orville (1983), Bulk parameterization of the snow field in a cloud model, J. Appl. Meteorol., 22, 1065–1092.

[jame20217-bib-0040] Lock, A. , A. Brown , M. Bush , G. Martin , and R. Smith (2000), A new boundary layer mixing scheme. Part I: Scheme description and single‐column model tests, Mon. Weather. Rev., 128, 3187–3199.

[jame20217-bib-0041] Mapes, B. , and X. Wu (2001), Notes and correspondence. Convective eddy momentum tendencies in long cloud‐resolving model simulations, J. Atmos. Sci., 58, 517–526.

[jame20217-bib-0042] Mapes, B. E. , and R. A. Houze Jr. (1995), Diabatic divergence profiles in western Pacific mesoscale convective systems, J. Atmos. Sci., 52, 1807–1828.

[jame20217-bib-0043] Noh, Y. , W. Cheon , S. Hong , and S. Raasch (2003), Improvement of the k‐profile model for the planetary boundary layer based on large eddy simulation data, Boundary Layer Meteorol., 107, 401–427.

[jame20217-bib-0044] Oueslati, B. , and G. Bellon (2013), Convective entrainment and large‐scale organization of tropical precipitation: Sensitivity of the CNRM‐CM5 hierarchy of models, J. Clim., 26, 2931–2946.

[jame20217-bib-0045] Pauluis, O. , and S. Garner (2006), Sensitivity of radiative‐convective equilibrium simulations to horizontal resolution, J. Atmos. Sci., 63, 1910–1923.

[jame20217-bib-0046] Pergaud, J. , V. Masson , S. Malardel , and F. Couvreux (2009), A parameterization of dry thermals and shallow cumuli for mesoscale numerical weather prediction, Boundary Layer Meteorol., 132, 83–106.

[jame20217-bib-0047] Petch, J. , and M. Gray (2001), Sensitivity studies using a cloud‐resolving model simulation of the tropical west Pacific, Q. J. R. Meteorol. Soc., 127, 2287–2306.

[jame20217-bib-0048] Petch, J. , P. Blossey , and C. Bretherton (2008), Differences in the lower troposphere in two‐and three‐dimensional cloud‐resolving model simulations of deep convection, Q. J. R. Meteorol. Soc., 134, 1941–1946.

[jame20217-bib-0049] Pinty, J. , and P. Jabouille (1998), A mixed‐phase cloud parameterization for use in mesoscale non hydrostatic model: Simulations of a squall line and of orographic precipitations, in *Conference on Cloud Physics*, pp. 217–220, Am. Meteorol. Soc., Everett, Wash.

[jame20217-bib-0050] Piriou, J.‐M. , J.‐L. Redelsperger , J.‐F. Geleyn , J.‐P. Lafore , and F. Guichard (2007), An approach for convective parameterization with memory: Separating microphysics and transport in grid‐scale equations, J. Atmos. Sci., 64, 4127–4139.

[jame20217-bib-0051] Raymond, D. , and S. Sessions (2007), Evolution of convection during tropical cyclogenesis, Geophys. Res. Lett., 34, L06811, doi:10.1029/2006GL028607.

[jame20217-bib-0052] Raymond, D. , and X. Zeng (2005), Modelling tropical atmospheric convection in the context of the weak temperature gradient approximation, Q. J. R. Meteorol. Soc., 131, 1301–1320.

[jame20217-bib-0053] Raymond, D. J. , S. L. Sessions , A. H. Sobel , and Ž. Fuchs (2009), The mechanics of gross moist stability, J. Adv. Model. Earth. Syst., 1, 9, doi:10.3894/JAMES.2009.1.9.

[jame20217-bib-0054] Ricard, J. , and J. Royer (1993), A statistical cloud scheme for use in an AGCM, Ann. Geophys., 11, 1095–1115.

[jame20217-bib-0055] Robe, F. , and K. Emanuel (2001), The effect of vertical wind shear on radiative‐convective equilibrium states, J. Atmos. Sci., 58, 1427–1445.

[jame20217-bib-0056] Romps, D. (2012a), Weak pressure gradient approximation and its analytical solutions, J. Atmos. Sci., 69, 2835–2845.

[jame20217-bib-0057] Romps, D. M. (2012b), Numerical tests of the weak pressure gradient approximation, J. Atmos. Sci., 69, 2846–2856.

[jame20217-bib-0058] Rutledge, S. A. , and P. V. Hobbs (1984), The mesoscale and microscale structure and organization of clouds and precipitation in midlatitude cyclones. XII: A diagnostic modeling study of precipitation development in narrow cold‐frontal rainbands, J. Atmos. Sci., 41, 2949–2972.

[jame20217-bib-0059] Schmidt, G. A. , et al. (2014), Configuration and assessment of the GISS modele2 contributions to the CMIP5 archive, J. Adv. Model. Earth. Syst., 6, 141–184, doi:10.1002/2013MS000265.

[jame20217-bib-0060] Sessions, S. , S. Sugaya , D. Raymond , and A. Sobel (2010), Multiple equilibria in a cloud‐resolving model using the weak temperature gradient approximation, J. Geophys. Res., 115, D12110, doi:10.1029/2009JD013376.

[jame20217-bib-0061] Shaevitz, D. , and A. Sobel (2004), Implementing the weak temperature gradient approximation with a full vertical structure, Mon. Weather Rev., 132, 662–669.

[jame20217-bib-0062] Shutts, G. , and M. Gray (1994), A numerical modelling study of the geostrophic adjustment process following deep convection, Q. J. R. Meteorol. Soc., 120, 1145–1178.

[jame20217-bib-0063] Skamarock, W. C. , J. B. Klemp , J. Dudhia , D. O. Gill , D. M. Barker , W. Wang , and J. G. Powers (2008), A description of the advanced research WRF version 3, technical report, DTIC Document.

[jame20217-bib-0064] Sobel, A. , and G. Bellon (2009), The effect of imposed drying on parameterized deep convection, J. Atmos. Sci., 66, 2085–2096.

[jame20217-bib-0065] Sobel, A. , and C. Bretherton (2000), Modeling tropical precipitation in a single column, J. Clim., 13, 4378–4392.

[jame20217-bib-0066] Sobel, A. , G. Bellon , and J. Bacmeister (2007), Multiple equilibria in a single‐column model of the tropical atmosphere, Geophys. Res. Lett., 34, L22804, doi:10.1029/2007GL031320.

[jame20217-bib-0067] Swann, H. (1998), Sensitivity to the representation of precipitating ice in CRM simulations of deep convection, Atmos. Res., 47–48, 415–435.

[jame20217-bib-0068] Tao, W. , J. Simpson , C. Sui , C. Shie , B. Zhou , K. Lau , and M. Moncrieff (1999), Equilibrium states simulated by cloud‐resolving models, J. Atmos. Sci., 56, 3128–3139.

[jame20217-bib-0069] Tiedtke, M. (1989), A comprehensive mass flux scheme for cumulus parameterization in large‐scale models, Mon. Weather Rev., 117, 1779–1800.

[jame20217-bib-0070] Tiedtke, M. (1993), Representation of clouds in large‐scale models, Mon. Weather Rev., 121, 3040–3061.

[jame20217-bib-0071] Tompkins, A. (2000), The impact of dimensionality on long‐term cloud‐resolving model simulations, Mon. Weather Rev., 128, 1521–1535.

[jame20217-bib-0072] Tompkins, A. , and G. Craig (1998), Radiative‐convective equilibrium in a three‐dimensional cloud‐ensemble model, Q. J. R. Meteorol. Soc., 124, 2073–2097.

[jame20217-bib-0073] Voldoire, A. , et al. (2013), The CNRM‐CM5.1 global climate model: Description and basic evaluation, Clim. Dyn., 40, 2091–2121.

[jame20217-bib-0074] Wang, S. , and A. Sobel (2011), Response of convection to relative sea‐surface temperature: Cloud‐resolving simulations in two and three dimensions, J. Geophys. Res., 116, D11119, doi:10.1029/2010JD015347.

[jame20217-bib-0075] Wang, S. , and A. Sobel (2012), Impact of imposed drying on deep convection in a cloud‐resolving model, J. Geophys. Res., 117, D02112, doi:10.1029/2011JD016847.

[jame20217-bib-0076] Wang, S. , A. H. Sobel , and Z. Kuang (2013), Cloud‐resolving simulation of TOGA‐COARE using parameterized large‐scale dynamics, J. Geophys. Res., 118, 6290–6301, doi:10.1002/jgrd.50510.

[jame20217-bib-0077] Weill, A. , et al. (2003), Toward a better determination of turbulent air‐sea fluxes from several experiments, J. Clim., 16, 600–618.

[jame20217-bib-0078] Wilson, D. R. , and S. P. Ballard (1999), A microphysically based precipitation scheme for the UK meteorological office unified model, Q. J. R. Meteorol. Soc., 125, 1607–1636.

[jame20217-bib-0079] Wilson, D. R. , A. C. Bushell , A. M. Kerr‐Munslow , J. D. Price , and C. J. Morcrette (2008), PC2: A prognostic cloud fraction and condensation scheme. I: Scheme description, Q. J. R. Meteorol. Soc., 134, 2093–2107.

[jame20217-bib-0080] Yano, J.‐I. , and M. Bonazzola (2009), Scale analysis for the large‐scale tropical atmospheric dynamics, J. Atmos. Sci., 66, 159–172.

[jame20217-bib-0081] Yao, M.‐S. , and Y. Cheng (2012), Cloud simulations in response to turbulence parameterizations in the GISS model E GCM, J. Clim., 25, 4963–4974.

